# Together, We Can Make This Place Our Home: Civic Engagement Among Asian Immigrants

**DOI:** 10.1093/geroni/igaa057.327

**Published:** 2020-12-16

**Authors:** Cindy Bui, Kyungmin Kim, Qian Song, Yuri Jang

**Affiliations:** 1 University of Massachusetts Boston, Boston, Massachusetts, United States; 2 University of Southern California, Los Angeles, California, United States

## Abstract

Civic engagement is an important dimension of age-friendly communities but has been understudied among Asian immigrant groups. While research has attributed greater civic engagement among immigrants to acculturation factors, the influence of acculturation may be conditioned upon Asian immigrants’ social network and place attachment to their city. We used data from the Asian American Quality of Life survey to analyze civic engagement activity (e.g., City council meeting, voting in a City election) among a diverse sample of middle-aged and older Asian immigrants in Austin, Texas (N = 994). 34.5% of the sample had participated in at least one civic engagement activity in the past 12 months. We examined how such civic engagement is associated with acculturation factors, and further examined whether one’s friend network and perception of their city moderated the association. We found that number of years lived in the U.S., familiarity with mainstream American culture, and number of friends in one’s social network were positively related to civic engagement activity. Furthermore, we found that the association between years lived in the U.S. and civic engagement was more pronounced for immigrants with larger friend networks; the association between familiarity with American culture and civic engagement was more pronounced for immigrants with more positive perceptions of the city. These findings highlight that acculturation may not operate alone in civic engagement among Asian immigrants. Rather, it may also be important to create opportunities for Asian immigrants to feel connected to their community and build meaningful friend networks to encourage civic engagement.

